# Critical Review on Zeolite Clinoptilolite Safety and Medical Applications *in vivo*

**DOI:** 10.3389/fphar.2018.01350

**Published:** 2018-11-27

**Authors:** Sandra Kraljević Pavelić, Jasmina Simović Medica, Darko Gumbarević, Ana Filošević, Nataša Pržulj, Krešimir Pavelić

**Affiliations:** ^1^Department of Biotechnology, Centre for High-Throughput Technologies, University of Rijeka, Rijeka, Croatia; ^2^General Hospital Pula, Pula, Croatia; ^3^Computer Science Department, University College London, London, United Kingdom; ^4^Juraj Dobrila University of Pula, Pula, Croatia

**Keywords:** zeolite, clinoptilolite, toxicology, immunostimulation, antioxidant properties

## Abstract

Unique and outstanding physical and chemical properties of zeolite materials make them extremely useful in a variety of applications including agronomy, ecology, manufacturing, and industrial processes. Recently, a more specific application of one naturally occurring zeolite material, clinoptilolite, has been widely studied in veterinary and human medicine. Due to a number of positive effects on health, including detoxification properties, the usage of clinoptilolite-based products *in vivo* has increased enormously. However, concerns have been raised in the public about the safety of clinoptilolite materials for *in vivo* applications. Here, we review the scientific literature on the health effects and safety in medical applications of different clinoptilolite-based materials and propose some comprehensive, scientifically-based hypotheses on possible biological mechanisms underlying the observed effects on the health and body homeostasis. We focus on the safety of the clinoptilolite material and the positive medical effects related to detoxification, immune response, and the general health status.

## Chemical Properties and Biological Application of Natural Zeolite Clinoptilolite

**FIGURE 1:**
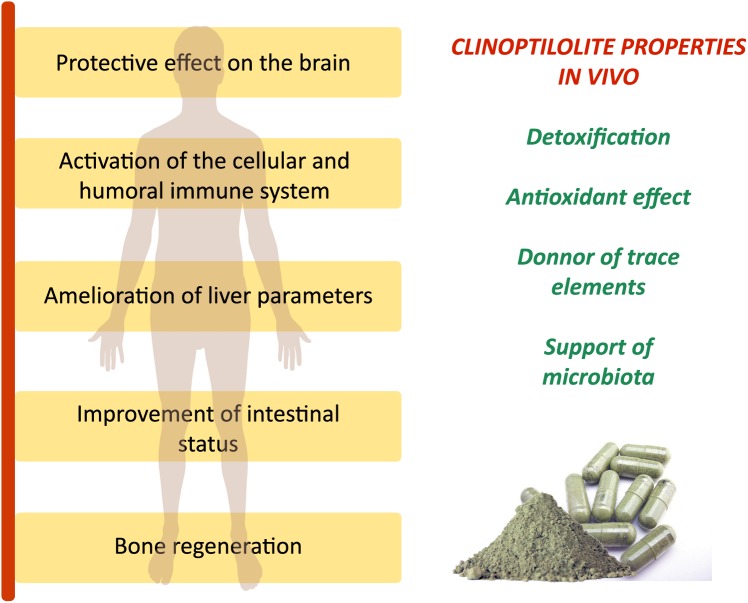
The generally accepted and studied clinoptilolite effects on the human body *in vivo*. Observed clinically relevant effects on organs and systems for different clinoptilolite materials *in vivo* are due to major clinoptilolite properties: detoxification, antioxidant effect, release of trace elements, and positive influence on the microbiota status in the intestine as described in Table 1. These effects were documented in animals and humans for clinoptilolite material used as supplementation to regular diet in a powdered form.

Zeolites possess unique and outstanding physical and chemical properties. These characteristics make them very useful in a variety of applications including agronomy, ecology, certain manufacturing, industrial processes, medicine, and cosmetics. Recently, the application of a specific natural zeolite material, clinoptilolite, has been documented in veterinary and human medicine. Subsequently, the market of clinoptilolite-based products for use *in vivo* has been continuously growing (Figure 1) (Pavelić and Hadžija, 2003).

The name ‘zeolite’ originates from the Greek words ‘zeo’(to boil) and ‘litos’ (a stone). The current nomenclature and classification of zeolite materials has been provided by the Structure Commission of the International Zeolite Association that identifies each material based on their framework with a three-letter mnemonic code; for instance, natural zeolite clinoptilolite is denoted as HEU (Baerlocher et al., 2007).

By origin, zeolites can be natural or synthetic materials. They are aluminosilicate minerals with rigid anionic frameworks containing well-defined channels and cavities. These cavities contain metal cations, which are exchangeable, or they may also host neutral guest molecules that can also be removed and replaced. The majority of natural zeolites are of volcanic origin and have a general formula, M2/n:Al_2_O_3_:xSiO_2_:yH_2_O, where M stands for the extra-framework cation (Bogdanov et al., 2009). The mineral structure is based on AlO_4_ and SiO_4_ tetrahedra, which can share 1, 2, or 3 oxygen atoms, so there is a wide variety of possible structures as the network is extended in three dimensions. This unique structural feature is a basis for their well-known microporous structure. Based on the pore size and absorption properties, zeolites are among the most important inorganic cation exchangers and are used in industrial applications for water and waste water treatment, catalysis, nuclear waste, agriculture, animal feed additives, and in biochemical applications (Bogdanov et al., 2009).

**FIGURE 2:**
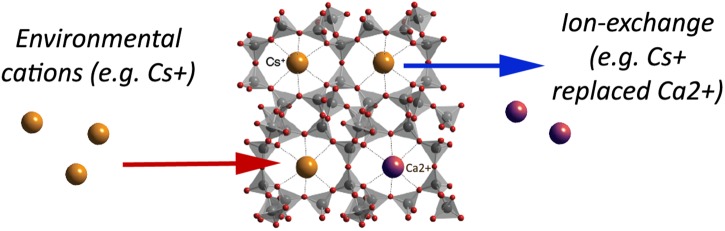
A simplified schematic of the clinoptilolite structure: linked SiO_4_ tetrahedra and pores with metal cations available for ion-exchange with environmental cations (e.g., caesium, Cs^+^) that are consequently trapped into the clinoptilolite pores. As naturally occurring clinoptilolite comes with pre-loaded cations (e.g., calcium, Ca^2+^), ion-exchange may occur depending on the ion-exchange capacity and cation affinity of the material, as well as on physical properties of the surrounding environment. In the herein presented simplified example, Cs^+^ enters in the zeolite pores instead of Ca^2+^ (adapted from http://www.chemtube3d.com/solidstate/SS-Z-Clinoptilolite.htm Creative Commons Attribution-Non-commercial-Share Alike 2.0 UK: England and Wales License). A detailed explanation of the clinoptilolite structure is given in the Database of Zeolite Structures (http://europe.iza-structure.org/IZA-SC/ftc_table.php).

The variety of zeolites’ application is indeed a consequence of their porous structure: pores form negatively charged channels and cavities, which are occupied with positively charged alkali, and alkali earth monovalent (i.e., Na^+^, K^+^), and divalent (i.e., Ca^2+^) ions, OH-groups or H_2_O molecules, which can be easily exchanged by other molecules and cations from the surroundings (Figure 2). It is logical then, that the final Si/Al ratio in a zeolite determines the ion exchange capacity and attraction of cations that come to reside inside the pores and channels (Mumpton, 1999; Canli et al., 2013a).

Besides metal cations and water resident in zeolites’ cavities and pores, other molecules and cationic groups may be accommodated as well, such as, for instance, ammonia, and nitrate ions, all of which are bound to different zeolites at different affinity levels (Gaikwad and Warade, 2014). For example, selectivity alignments of the zeolite clinoptilolite cation exchange have been given as Ba^2+^> Cu^2+^, Zn^2+^> Cd^2+^, Sr^2+^> Co^2+^ by Blanchard et al. (1984), as Pb^2+^> Cd^2+^> Cs^+^> Cu^2+^> Co^2+^> Cr^3+^> Zn^2+^> Ni^2+^> Hg^2+^ by Zamzow et al. (1990), or as Co^2+^> Cu^2+^> Zn^2+^> Mn^2+^ by Erdem et al. (2004).

The mineral assemblies of the most common zeolite occurrences in nature are clinoptilolite- and mordenite-containing tuffs, in which the zeolite clinoptilolite and mordenite content is high (80% and over). It may appear with the aluminum phyllosilicate clay smectite (bentonite) and accompanying phases present in lower percentages cristoballite, calcite, feldspar, and quartz. However, other types of zeolites (e.g., phillipsite, chabazite) and clay minerals may dominate the mineral tuff assemblage, and properties of such materials may vary in the widest sense with respect to the final mineral content (Cejka, 2005).

The widely tested zeolite suitable for medical applications *in vivo* is the clinoptilolite tuff but the mordenite tuff was also studied by Selvam et al. (2014) So far the word ‘zeolite’ has been used in the literature for different types of zeolites, tuffs, and clays. For example, both clinoptilolite and clay materials may be used for ion-exchange reactions. Still, their structural properties and toxicology profiles may be different (Maisanaba et al., 2015). The structure of mineral clays is, for instance, organized in layers (sheets), while clinoptilolite has tetrahedra arranged in a way that they form large amounts of pore space in the crystals. Different physical-chemical properties between clinoptilolite and clays, e.g., kaolinite were documented accordingly in the literature (Ghiara et al., 1999; Miranda-Trevino and Coles, 2003; Payra and Dutta, 2003; Hecht, 2005; Svoboda and Šulcová, 2008; Bibi, 2012; Dimowa et al., 2013; Jurkić et al., 2013). For example, the kaolinite structure may change during the ion-exchange processes due to the displacement of H^+^ ions or due to the swelling of the structure as a consequence of Pb, Zn, or Cd cations absorption, which is opposite to the clinoptilolite constancy during ion-exchange process (Miranda-Trevino and Coles, 2003).

Clinoptilolite shares a high structural similarity with the zeolite heulandite (they are isostructural) and it is distinguished from helaundite by a higher silicon to aluminum ratio in favor to silicon, where Si / Al > 4.0 and (Na + K) > (Ca + Sr + Ba). The thermal behavior of clinoptilolite and heulandite is also different. The clinoptilolite structure is still not destroyed after 12 h of heating at 750°C, whereas the heulandite structure is destroyed after 12 h at 450°C (Ghiara et al., 1999). This structural stability is an essential element for *in vivo* applications.

For instance, a synthetic material known as Zeolite A, used widely for ion-exchange in industrial processes, has the framework composition with a high Al content and the molar ratio of Si/Al of almost 1. This is indeed the highest aluminum content possible in tetrahedral alumosilicate frameworks (Payra and Dutta, 2003). In Zeolite A, the Al-framework is balanced out by the maximum number of cation exchange sites; it has high cation contents and superior exchange capacities. However, it is not appropriate for *in vivo* applications since, similar to other low-silica zeolites, zeolite A is unstable in acids. In contrast, zeolites with higher silica content, such as clinoptilolite, are stable in acids (Payra and Dutta, 2003).

We present a comprehensive review of clinoptilolite applications in veterinary and human medicine. We consider all of the above clinoptilolite properties and propose its mechanisms of action *in vivo* (summarized in Table 1) and propose some comprehensive, scientifically-based hypotheses on possible biological mechanisms underlying observed effects on the health and body homeostasis.

**Table 1:** Documented properties and effects of clinoptilolite relevant for biomedical applications and effects in animals and humans.

Clinoptilolite properties	Clinoptilolite effects
Cation exchange capacity (Mumpton, 1999; Pavelić et al., 2001a; Pavelić and Hadžija, 2003)	Detoxicant, mineral donor (EFSA Panel on Additives and Products or Substances used in Animal Feed, 2013; Jurkić et al., 2013; Exley, 2016; Kraljević Pavelić et al., 2017)
Molecular sieve (size and shape selectivity) (Mumpton, 1999; Pavelić and Hadžija, 2003)	Impact on the intestine status (Yao et al., 2016)
Selective adsorption of water (Kotova et al., 2016)	Immunomodulation (Ivkovic et al., 2004; Montinaro et al., 2013)
Removal of ammonia ions and uremic toxins (urea, uric acid, creatinine, *p*-cresol, indoxyl sulfate) (Demir et al., 2002; Auerbach et al., 2003; Sprynskyy et al., 2005; Joughehdoust and Manafi, 2008; Zabochnichka-Światek and Malinska, 2010)	Effect on pathogens and microbiota (Zarkovic et al., 2003; Saribeyoglu, 2011; Prasai et al., 2016)
Reversible binding of small molecules (Pavelić and Hadžija, 2003)	Enzyme mimetics, metaloenzyme mimicry (Herron, 1989)
Biosensors (Soldatkin et al., 2015)	Antitumor adjuvant (Wesley, 1996; Krewski et al., 2009)
Drug carrier/delivery (Hayakawa et al., 2000; Bonferoni et al., 2007)	Vaccine adjuvant (Garces, 1999)


## Use of Clinoptilolite in Veterinary and Human Medicine

Studies performed in the last decades showed a high potency of clinoptilolite in diverse medical applications *in vitro* and *in vivo* (Jurkić et al., 2013). A large number of documented positive clinoptilolite medical effects were attributed to basic clinoptilolite material properties, in particular, to reversible ion-exchange and adsorption capacity (Mumpton, 1999; Pavelić et al., 2001a; Jurkić et al., 2013). This central clinoptilolite characteristic related to elimination of toxic agents, which may be seen as a support to the ‘body homeostasis,’ could be widely exploited in a number of medical applications. For instance, a high affinity of clinoptilolite toward ammonia was proven in different systems for elimination of ammonia from water (Demir et al., 2002; Sprynskyy et al., 2005; Zabochnichka-Światek and Malinska, 2010). This is why clinoptilolite has widely been used for years in animal production as an additive to animal feed, or for the removal of ammonia in animal manure (Auerbach et al., 2003). This ammonia affinity is an interesting feature for medical applications in humans as well. For example, detrimental roles of the end-products of protein fermentation, such as ammonia, have been recognized on the colonic microbiota and epithelial health, in particular on the colonocytes life span and function (Hughes and Magee, 2000; Yao et al., 2016; Hamid Said, 2018).

The excessive production of ammonia, but also of other gaseous products, including CO_2_ and H_2_S, may occur as a consequence of protein-rich or imbalanced diets, or in diverse pathogeneses where excessive protein fermentation occurs, including irritable bowel syndrome, ulcerative colitis, and colorectal carcinogenesis (Hughes and Magee, 2000; Yao et al., 2016). Clinoptilolite has a high affinity toward ammonium and may prove useful in these cases as an adjuvant to the standard therapy (Yao et al., 2016). From this perspective, clinoptilolite was evaluated in a recent trial performed on aerobically trained subjects (Lamprecht et al., 2015). In this study, endurance-trained subjects were recruited and supplemented with a clinoptilolite/dolomite/maca-based product (Panaceo Sport^®^). Athletes, indeed, often report intestinal symptoms including nausea, stomach and intestinal cramps, vomiting, and diarrhea. These symptoms may be a consequence of typical athletes’ diets with high protein content, as in such circumstances excessive protein fermentation may occur and is accompanied by higher ammonia release in the intestine as well. These subjects also have increased intestinal wall permeability. A well-known and complex relationship between exercise and oxidative stress depends on many diverse factors. For instance, regular moderate exercise increases the resistance against oxidative stress, while acute and vigorous exercise can generate free radicals in excess. Consequences of exercise at exhaustion levels include increased number of leukocytes due to the damage of muscle fibers and connective tissue (Morillas-Ruiz and Hernández-Sánchez, 2015) as well as elevated lipid-peroxidation marker MDA in the plasma (Pingitore et al., 2015). It is, therefore, not surprising that a number of professional athletes show gastrointestinal symptoms, which may result in medical problems, infections, and autoimmune disease (Waterman and Kapur, 2012; Oliveira et al., 2014). Interestingly, the supplementation with Panaceo Sport positively influenced the intestinal wall integrity, which was witnessed through decreased concentrations of the tight junction modulator zonulin, a marker of increased intestinal permeability (Lamprecht et al., 2015).

Other studies on detoxification properties of clinoptilolite materials *in vivo* performed so far have mainly been done on animals and they provide strong evidence on alleviating effects during exposure to different toxicants upon clinoptilolite supplementation. For instance, a prolonged consumption of water with increased nitrate levels by dairy cattle is known to impair protein metabolism and glucose utilization. In these cows, dietary administration of clinoptilolite alleviated the nitrate burden to the body and reduced the negative systemic effects of nitrates (Katsoulos et al., 2015). Similarly, a dietary mixture containing 3% of a clinoptilolite-based product showed an increase in the nitrogen excretion in feces and a decrease in the nitrogen excretion in urine in growing pigs. Importantly, no effects on the protein retention values were observed and the protein deposition was not altered (Poulsen and Oksbjerg, 1995; Laurino and Palmieri, 2015).

Moreover, clinoptilolite incorporated into the diet may be effective in fighting mycotoxins by direct absorption. Affinity toward aflatoxins, zearalenone, ochratoxin, and the T2 toxin was proven *in vitro* in the presence of aminoacids and vitamins, where the latter were not absorbed by the clinoptilolite material (Tomasevic-Canovic et al., 1996). The specificity for aflatoxin M1 was also shown *in vivo*, and the dietary administration of clinoptilolite, especially of the material with the smallest particle size at the rate of 200 g per cow per a day, effectively reduced milk aflatoxin M1 concentration in dairy cattle (Katsoulos et al., 2016).

It is important to note that the supplementation with clinoptilolite in dairy cows may have additional benefits, such as the reduction of parturient paresis. A study by Katsoulos et al. (2005a), for instance, showed that the clinoptilolite supplementation reduced its incidence and did not affect serum concentrations of total calcium, phosphate, magnesium, potassium, and sodium. This veterinary application showed that mineral levels in the blood were not affected by clinoptilolite supplementation which may be relevant for human applications as well. Indeed, the demand for healthier food products and a balanced diet is being increasingly recognized as a central paradigm for the preservation of the body’s homeostasis and health. Moreover, it is widely known that the contamination of poultry by food-borne pathogens is considered a major problem in the poultry industry. This is why antibiotics are standardly used in poultry meat production. Such a wide use of antibiotics in poultry, but also in the production of other meat, has recently been accepted as a major cause for development of antibiotic-resistant bacteria (Aminov and Mackie, 2007). New, natural possibilities for improvement of animal health in meat production have therefore been widely discussed (Diaz-Sanchez et al., 2015) and clinoptilolite may be a natural alternative.

For instance, clinoptilolite has been tested as a possible supplementation to broilers feed as an alternative to antibiotics for: (1) the control of the total flora at broiler farms, where clinoptilolite supplementation showed a positive effect on the total flora, a parameter often used in the evaluation of the gastrointestinal health status in poultry (Mallek et al., 2012), as well as on the performance of production and organoleptic parameters, especially on the increase of omega-3 fatty acid levels in eggs (Mallek et al., 2012); (2) the improvement of the antioxidant capacity in broilers where the supplementation of clinoptilolite materials increased the activities of glutathione peroxidase, catalase, total SOD, and the total antioxidant capacity (Wu Y. et al., 2013); (3) the reduction of mycotoxin effects on broilers health, where the number of aflatoxin-affected broilers, or the number of severe lesions in the liver of chickens, was reduced in the clinoptilolite-supplemented group (Ortatatli and Oğuz, 2001). All these documented effects are due to the clinoptilolite capacity to adsorb harmful substances in the gastrointestinal tract that are not confined only to micotoxins and ammonia but include heavy metals and organic compounds as well.

Indeed, different studies have shown that clinoptilolite materials provide direct detoxifying performance *in vivo*. For instance, in lead-intoxicated mice, a clinoptilolite sorbent KLS-10-MA decreased the lead accumulation in the intestine by more than 70% (Beltcheva et al., 2012, 2015). Moreover, in rats exposed to organophosphate poisoning, zeolite tuff containing 61% of clinoptilolite and added 5 min prior to intoxication at dosage 1 g/kg, proved efficient in the restoration of cholinesterase activity in the brain, liver, spleen, femoral muscle, heart, stomach, duodenum, colon, and erythrocytes of intoxicated animals (Mojzis et al., 1994). Two possible ways of binding organophosphates may be envisaged. One is the esterification reaction of the free OH moiety and the carboxyl functional group of the acid. The second option is through adsorption by forming a dipol–dipol interaction between the polar channel and/or the zeolite surface and fluorine, or on the acid. It can generally be stated that clinoptilolite loaded with potential toxicants in the intestine is then excreted along with toxicants (EFSA Panel on Additives and Products or Substances used in Animal Feed, 2013).

It seems that this detoxifying effect may have additional systemic effects. The role of clinoptilolite has been recognized in medical applications, where its usage in zootechnology and veterinary medicine has provided strong evidence on improvement of pets’ fitness and efficiency in the removal of numerous harmful substances from the organism, including radioactive elements, mycotoxins, and poisons (Laurino and Palmieri, 2015). In addition, EDTA and clinoptilolite supplementation exerted a protective effect on the brain tissue of mice intoxicated with lead by inducing antioxidant mechanisms and greater activity levels of catalase, SOD, glutathione peroxidase, and glutathione (Basha et al., 2013). Moreover, a study in humans showed the ability of tribomechanically micronized clinoptilolite to decrease the absorption of ingested ethanol by reducing blood alcohol levels at a dose of 5 g (Federico et al., 2015). If the clinoptilolite-containing product dosage is lower or if it is not administered at the time of alcohol consumption, this effect may not be visible as shown by Gandy et al. (2015) where clinoptilolite still proved highly efficient in the reduction of veisalgia symptoms and signs up to 40–50%.

In addition, clinoptilolite has interesting antioxidant, hemostatic, and anti-diarrheic properties that may be exploited in human medicine, especially as adjuvants to standard therapies (Pavelić and Hadžija, 2003). However, the number of clinical studies with clinoptilolite materials on humans is still low, and the previously described immunomodulatory, anticancer, and antioxidant effects of clinoptilolite *in vivo* should be studied in more detail.

Even though the efficacy and potential of clinoptilolite materials in medicine seems high, questions have been raised on to possible clinoptilolite effects on physiologically relevant elements, i.e., micronutrients and trace elements, or effects on important processes in the organism. The results published thus far show that clinoptilolite does not affect the homeostasis of trace elements and micronutrients, but acts rather selectively on heavy-metals and toxicants. For instance, clinoptilolite-treated dairy goats showed no changes in serum concentrations of fat-soluble vitamins, macro-elements, and trace elements, or activities of hepatic enzymes. In addition, clinoptilolite supplementation improved milk fat percentage and milk hygiene (Katsoulos et al., 2009). No effects of clinoptilolite on physiological mineral levels have been observed in cows (Katsoulos et al., 2005a; Valpotić et al., 2017).

## Zeolites Effects on Oxidative Stress and Immune System

In aerobic organisms, production of small quantities of ROS, including peroxides, superoxides, hydroxyl radicals, and singlet oxygen, occurs continuously (Hayyan et al., 2016). A controlled production of ROS is indeed essential to the body’s homeostasis (Covarrubias, 2008), while an excessive production of ROS is known to cause damage to the DNA, proteins, and lipids (Gulam and Ahsan, 2006). Some ROS are produced endogenously, while others are derived exogenously, such as those formed by ionizing radiation. The endogenous sources of ROS are the mitochondria, cytochrome P450 metabolism, peroxisomes, and inflammatory cell activation (Inoue et al., 2003). For example, mitochondria-produced ROS are the superoxide anion ($O_{2}^{•-}$), hydrogen peroxide (H_2_O_2_), and the hydroxyl radical (⋅OH). Other routes and factors may induce ROS in the organism as well, such as ROS produced through the activity of xanthine oxidase, in reactions of hypoxanthine to xanthine and xanthine to uric acid conversions, where molecular oxygen is reduced to superoxide anion, followed by a generation of hydrogen peroxide (Valko et al., 2004). It is understood that homeostasis in normal cells includes a balance between ROS production and antioxidant defense activity. Indeed, antioxidant mechanisms in the human body, which are the main regulators of ROS levels, are based on enzyme and non-enzyme systems. Enzyme systems rely mainly on SOD, catalase, peroxiredoxin (Prx), thioredoxins (Trx), and glutathione (GSH) enzymes’ activity, while non-enzymatic systems comprise flavonoids, vitamin A, vitamin C, vitamin E, and melatonin (Rahman, 2007). In addition to these antioxidant systems inherent to the body, other exogenous antioxidants are important in the regulation of constant body’s ROS homeostasis as well. For example, dietary compounds are highly important for elimination of excessive ROS caused by external stimuli and include, for instance, carotenoids, tocopherols, bioflavonoids, anthocyanins, and phenolic acid (Smilin Bell Aseervatham et al., 2013). When ROS production exceeds antioxidant capacity, we usually perceive the process as “oxidative stress” that leads to organic damage. Increased oxidative damage to cells and tissues and the modulation of the ROS-regulated signaling pathways have recently been acknowledged in the pathogenesis of a wide number of diseases, including obesity, atherosclerosis, heart failure, uremic cardiomyopathy, kidney pathologies, hypertension, neurological disease, and cancer (Chen et al., 2016; Miranda-Díaz et al., 2016; Patel, 2016; Srikanthan et al., 2016; Ding et al., 2017). It should be noted that for a proper functioning of the body, antioxidant defenses, co-factors, or molecules that activate enzymes by binding to their catalytic sites are also required. In case of antioxidant enzymes, these co-factors may include the coenzyme Q10, vitamins B1 and B2, carnitine, selenium, and often transition metals Cu, Mn, Fe, and Zn (Khalid, 2007). Recently, a preliminary efficacy study performed on patients with dyslipidemia has also shown a positive effect of clinoptilolite supplementation on lowering the total lipid count and LDL (low density lipoproteins), which may also be indirectly correlated with its general antioxidative effect (Cutovic et al., 2017).

Due to a certain amount of pre-loaded elements, it is possible to assume that clinoptilolite may positively affect the body’s metal homeostasis, including either the levels or the availability of some physiological metal ions that are pre-loaded in the material, on signal pathways responsible for the production of endogenous antioxidant enzymes. Still, no direct data supports these assumptions that may partially explain the observed effects on the oxidative stress defense mechanisms, which are visible as activation or restoration of activity and levels of natural antioxidant enzymes. Still, this effect should be evaluated along with factors such as, for example, the applied daily dosage, health status, or lifestyle. For example, in the study of Lamprecht et al. (2015), the daily dosage of 1.85 g clinoptilolite material supplementation did not show an effect on the measured redox markers in the blood of healthy athletes. Furthermore, interesting effects of clinoptilolite supplementation were documented in animals as well. In hepatectomized rats, for instance, common oxidative stress markers are induced upon trauma, including MDA in the plasma and liver tissue. When hepatectomized rats were supplemented with a micronized clinoptilolite preparation, ‘Froximun,’ MDA levels were significantly lower, while liver tissue antioxidant mechanisms were strengthened, as witnessed by a significantly higher activity of Cu-Zn SOD and GSH (Saribeyoglu, 2011). Also, in chicken, daily supplementation with a natural clinoptilolite, or a modified clinoptilolite, efficiently improved the antioxidant capacity by increasing the antioxidant enzyme activities in intestine mucosa and decreasing the free radical NO content and inducible nitric oxide synthase activity in the serum. Moreover, upon prolonged supplementation in chicken, both tested clinoptilolite materials increased the activities of glutathione peroxidase, catalase, total SOD, and the total antioxidant capacity (Wu Q.J. et al., 2013). Similarly, in doxorubicin treated mice, micronized clinoptilolite proved efficient in counteracting lipid peroxidation in the liver (Zarkovic et al., 2003).

An interesting effect of clinoptilolite was observed in fluoride-intoxicated rats (Madhusudhan et al., 2009). Fluoride is neurotoxic upon penetration through the blood–brain barrier during gestation and post-gestation periods. As a consequence of fluoride-intoxication, inhibition of antioxidant enzymes occurred in pups along with lipid peroxidation. Upon supplementation of pups with clinoptilolite, oxidative damage was restored and levels of GSH-Prx were substantially ameliorated in the cerebral cortex and medulla oblongata. Similar results were, however, observed in animals supplemented with vitamins E and C as well (Madhusudhan et al., 2009). In line with these results, it should also be hypothesized that clinoptilolite might have the potential to combat acute fluoride-intoxication in animals, as well as in humans. In the gastric juice, fluoride anions are converted into hydrofluoride acid. Such a weak hydrofluoride acid may form hydrogen bonds with the clinoptilolite framework and be eliminated from the body in the stool.

We believe that exact mechanisms of clinoptilolite effects on systemic restoration of homeostasis and increased antioxidant capacity are still not fully understood, as these effects are in our opinion probably connected both to general detoxifying effects occurring in the intestine, to immunomodulatory effects, or even to the release of physiologically-relevant cations from the clinoptilolite framework during the ion exchange process, e.g., Ca, Mn, Zn, and Mg, which are then readily available to the organism and the antioxidant mechanism. Similar indirect effects of clinoptilolite on the antioxidant mechanisms in the body were also observed in different pathologies and disease models. For instance, tribomechanically-micronized zeolite increased SOD activity in a transgenic mouse model of the Alzheimer disease in the hippocampus and cortex, while it concomitantly reduced Aβ (x-42) amyloid beta levels in the hippocampus (Montinaro et al., 2013). Moreover, zinc-bearing clinoptilolite proved to exert a protective effect on the performance and gut health of broilers against *S. pullorum* infection as well as to improve the SOD activity of ileal mucosa and reduced MDA contents of jejunual and ileal mucosa (Wang, 2012).

It is also possible that antibacterial and antiviral effects of clinoptilolite might be in correlation with immunomodulatory properties. For instance, in long-term supplementation with clinoptilolite, a decreased prevalence of *E. coli* carrying certain antimicrobial resistance and virulence genes was documented (Jahanbakhsh et al., 2015). An influence of natural clinoptilolite on *E. coli* was also documented in another study on broilers *in vivo* (Wu Y. et al., 2013). In this study, a beneficial effect on intestinal parameters was measured, which was hypothesized to be based on a direct effect on the microbial population in the intestine. While the total count of *E. coli* was significantly reduced, a rise of Lactobacillus acidophilus occurred in parallel (Zarkovic et al., 2003). Similarly, clinoptilolite supplementation of Enterex^®^, approved by the Cuban Drug Quality Control Agency, showed to be highly efficient in ameliorating diarrhea symptoms in several clinical studies on humans with acute diarrhea of different etiologies. Moreover, in cases where diarrhea symptoms were removed and the pathogenic agent was identified upon Enterex treatment antibiotics were additionally used to completely eliminate pathogenic bacteria from the intestinal lumen (Rodríguez-Fuentes et al., 1997). Therefore, this observed antidiarrheal activity may be in correlation with Enterex^®^ effect on certain pathogenic bacteria count or the microbiota status in general rather than with a direct antibacterial effect, which would have to be confirmed by additional studies. Recently, a positive effect of a potentiated clinoptilolite material (Absorbatox^®^) was also shown to reduce symptoms associated with endoscopically negative gastroesophageal reflux disease and non-steroidal anti-inflammatory drug-induced gastritis, where it significantly prevented mucosal erosion severity (Potgieter et al., 2014).

Similarly, antiviral properties for clinoptilolite *in vitro* were shown on the human adenovirus 5, herpes simplex virus type 1, and the human enteroviruses coxsackievirus B5 and echovirus 7 (Grce and Pavelić, 2005). This effect may probably be attributed to a direct adhesion of the viral particles on clinoptilolite *in vitro*, which then inhibits viral entrance in the cells and viral replication. Even though no *in vivo* studies on clinoptilolite antiviral activity have been published thus far, positive immunomodulatory effects have been observed in patients treated for immunodeficiency disorders. In a study performed by Ivkovic et al. (2004), a significant increase in specific immunity cell counts, B lymphocite CD19+, T-helper cells CD4+, and activated T-lymphocytes HLA-DR+ were observed in subjects treated with tribomechanically micronized clinoptilolite. This effect was accompanied by significantly decreased natural immunity NK CD56+ cell counts. Again, standard blood count parameters of patients remained within normal referent values (Ivkovic et al., 2004).

A hypothesis for the observed clinoptilolite immunomodulatory effects may be the modulation of body defense mechanisms toward ROS. Indeed, ROS induces cell and tissue damage when the inflammation is initiated as a mechanism for restoration of the body’s homeostasis. Any impairment of the host immune and inflammatory mechanisms in the long-term may cause other inflammatory disorders, e.g., chronic sinusitis, otitis media and osteomyelitis, or microbial overgrowth syndromes, such as bacterial vaginosis, or inflammatory bowel disorders. It is plausible, therefore, to assume that such disorders have the formation of biofilms in common due to the impaired immunological reaction of the host organism (Pincus, 2005). Indeed, previous studies have shown a link between the antioxidative effect and the stimulation of the immune system (Knight, 2000; Brambilla et al., 2008).

**FIGURE 3:**
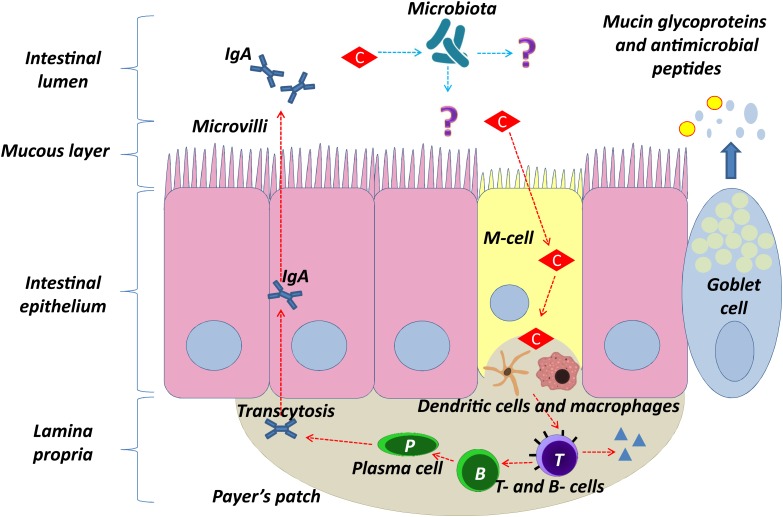
Proposed model of clinoptilolite positive immunomodulatory effect in the intestinal epithelium (denoted with red arrows) through interaction of clinoptilolite tuff particles with microfold cells (M-cells). Clinoptilolite tuff released particles are denoted by ‘C.’ M-cells are hypothesized to transport luminal clinoptilolite tuff released particles across the epithelial barrier and present them to immunological cells (e.g., dendritic cells) in the lamina propria and the Peyer’s patches. The latter are rich in T cells, macrophages, and clinoptilolite- activated IgA secreting B and plasma cells. The single layer of the intestinal epithelium is protected by mucus containing mucin glycoproteins where immunoglobulin A (IgA) and antimicrobial peptides prevent interaction of microbiota with the cell surface. Question marks (?) and blue arrows denote still unknown interactions of clinoptilolite with microbiota and microbiota with the lumen and epithelia.

Clinoptilolite’s positive immunomodulatory effects in similar conditions may be due to the interactions of clinoptilolite particles in the intestine with microfold cells (M-cells) (Figure 3). M-cells are found in the GALT of Peyer’s patches, a rich lymphoid tissue that communicates with intestinal epithelial cells and the microbiome of the intestine by diverse immunomodulation processes as well as in the MALT of other parts of the gastrointestinal tract. These gastrointestinal cells are known to initiate mucosal immunity responses on the apical membrane of the M-cells and to allow the transport of microbes and particles across the epithelial cell layer from the gut lumen to the *lamina propria* where interactions with immune cells occur (Mabbott et al., 2013). While evaluating possible clinoptilolite immunomodulatory effects in the intestine, it should be emphasized that M-cells can uptake nano- and submicro-particles, which can probably induce changes in the redox homeostasis in a cell (Igarashi, 2015). These changes in the M-cells then affect the Peyers patches as well. It is important to note that M-cells apical and basolateral sides, which communicate with Peyers patches, are polarized (Society for Mucosal Immunology, 2012) and one may hypothesize that, due to this particular phenotype, M-cells retain clinoptilolite particles or silica particles released from the clinoptilolite material (tuff), which do not enter the blood system (Nizet et al., 2018) and act locally on this tissue. Contrary to M-cells, other cells in the intestine cannot perform macropinocytosis and therefore cannot absorb negatively charged clinoptilolite particles or silica particles released from the clinoptilolite material (tuff) due to their rich negatively-charged glycoprotein-polysaccharide covering, glycocalix (Egberts et al., 1984). Some probiotics’ metabolites, e.g., from the lactic acid bacteria, exert the same activating function on Peyers patches as we suggest for clinoptilolite particles or silica particles released from the clinoptilolite material (tuff) and improve intestinal wall integrity (Sung et al., 2016). Therefore, we propose that this clinoptilolite-induced M-cells’ communication with Peyer’s patches, as similarly shown by Pavelic et al. (2002), increases the immune response either through particle intake or microbiota effect as recently shown in dogs supplemented with the zeolite chabazite (Sabbioni et al., 2016), and in particular, stimulates IgA producing B lymphocytes (plasma cells), a defensive mechanism of the intestinal tract against pathogenic bacteria (Round and Mazmanian, 2009). In the paper by Nizet et al. (2018), however, (Egberts et al., 1984), no clinoptilolite particles were detected in the selected sections of the gut tissue. Even though the inspection of limited histopathological sections in this study cannot rule out the suggested hypothesis on clinoptilolite particles or silica particles released from clinoptilolite material (tuff) in activation of Peyer patches, experimental analysis of the observed local immunomodulatory effect should be conducted in more detail. Indeed, the microbiota-clinoptilolite interaction may also underlie the observed immunomodulatory mechanism as well. Indeed, a role of IgA was already described in the reduction of intestinal pro-inflammatory signaling and bacterial epitope expression as part of the innate immune mechanism that contributes to balancing antibodies’ negative impact on the micriobiota status (Round and Mazmanian, 2009). Evidence was provided on the role of cross-talking between the adaptive immune system and gut microbiota by selective generation of immune responses to bacteria that consequently stimulate the innate system and production of IgA. By means of this mechanism, the host can detect new bacterial types and ignore previously encountered bacteria in the intestine (Peterson et al., 2007). This immunomodulatory effect of clinoptilolite was speculated to be the so-called ‘silicate superantigen’ response. The superantigens generally encompass some bacterial exotoxins and viral products with a potent non-specific immuno-stimulatory effect on large T-cells fractions. This immunostimulation occurs upon simultaneous interaction of the superantigen with MHC class II molecules and T-cell receptors. Superantigens bind to the variable Vβ region of the T-cell receptor or to CD28 and do not follow the peptide-binding pattern. An incredibly heterogeneous T-cell clonal activation occurs upon binding and different cytokines are produced massively (Proft and Fraser, 2016). The superantigen-activated T-lymphocytes provoke the cellular immune response and also the humoral immune response, as postulated by Emmer et al. (2014) in multiple sclerosis pathogenesis as well. Lymphocytes stimulation by silicates, which also act as superantigens, was already shown for different silicate materials in *in vitro* conditions and this mechanism may underlie immunomodulation activity of clinoptilolite in the intestine as well (Ueki et al., 1994; Aikoh et al., 1998). Even though the exact mechanisms remain elusive, one may speculate that clinoptilolite silica or released silica acts as a superantigen that promotes the formation of IgA producing plasma cells, which is dependent on the presence of superantigen-reactive T cells. A similar superantigen effect was already observed in Peyer’s patches during milk-borne mouse mammary tumor virus infection (Cabrera et al., 2010). To our knowledge, no negative effects on immune cells or tissue were documented in the scientific literature so far. Also, we cannot rule out some other unrecognized immunomodulatory effects of clinoptilolite due to a direct interaction with human microbiome (Figure 3).

The majority of studies on clinoptilolite were done by using different, so-called activated materials to increase either the surface area or to improve the clinoptilolite general adsorption or the ion-exchange capacity. Activation may be performed either through chemical treatment, e.g., with an acid, by replacing stabilizing cations, or through mechanical modifications by means of different micronization methods, which may all increase the surface area and change the ion-exchange properties and adsorption capacity (Abdulkerim, 2012; Akimkhan, 2012; Canli et al., 2013b). In the paper by Kraljević Pavelić et al. (2017), it was specifically shown that different micronization methods change the clinoptilolite tuff properties by affecting the surface area, pore size, and silicon to aluminum ratio on the surface of the material. Moreover, hydrochloric acid (HCl) that is also present in the stomach may change clinoptilolite physical chemical properties and has been proven to enhance the clinoptilolite ion-exchange capacity for Cu^2+^ and Co^2+^ in a synthetic Cu-Co solution at concentrations relevant for the stomach *in vivo* (0.1 M) (MambaI et al., 2010). Still, the clinoptilolite ion-exchange effects *in vivo* are complex and cannot be linearly explained as they are not affected only by the environmental conditions (pH, temperature, etc.) but also by the material composition and cation affinity properties. In a recent article, Turkish clinoptilolite was activated with hydrogen peroxide, which acts as a weak acid, to improve Ni^2+^ ions removal from aqueous solutions (Çanli and Abali, 2016). The authors show changes on the clinoptilolite surface upon activation that resulted in an improved Ni-ions absorption. This is important, as hydrogen peroxide dissociates into hydrogen ion H^+^ and hydrogen peroxide radical ${\left({\mathrm{HO}}_{2}^{•}\right)}^{-}$, and during the acid-activation process H^+^ ions are brought to the negatively-charged species on the material surface. As a consequence, de-alumination of the surface occurs, which increases the Si/Al surface ratio and absorption capacity for metal cations. This is a well-known process in industrial applications, while for the *in vivo* applications it may also hold certain relevance. *In vivo*, the acid concentrations of the intestine are substantially lower than those used in industrial activation process. For instance, gastric acid in the stomach contains HCl at 0.05–0.1 M. In such an environment, a certain release of Al species from the clinoptilolite surface may well be hypothesized even though aluminum from the clinoptilolite materials does not enter the blood or accumulate in the body as shown in athletes supplemented with zeolite-clinoptilolite supplement (Lamprecht et al., 2015) or healthy rats supplemented with different clinoptilolite materials (Kraljević Pavelić et al., 2017) where aluminum released into systemic circulation was observed only in rats supplemented with synthetic zeolite A. The latter effect was attributed to the zeolite A lower stability in the acidic pH relevant for the human intestine in comparison to clinoptilolite materials. In this study, authors also proved that clinoptilolite materials were efficient in the removal of aluminum from aluminum chloride-intoxicated rats *in vivo*. These observations may be attributed to the clinoptilolite stability, the low bioavailability of Al species from water (around 0.1 to 0.4%), and the immediate precipitation of Al-species as non-soluble forms. Aluminum(III)-cation (Al^3+^) has a generally strong affinity for anions which promote its precipitation. The Al^3+^ in most situations seeks out complexing agents with oxygen-atom donor sites, such as carboxylate or phosphate groups, e.g., from food in the intestine. However, it should be noted that the aqueous coordination chemistry of Al^3+^, especially in the living systems, is rather complex due to the Al-complexes’ tendency to hydrolyze and form polynuclear species, which vary according to the pH condition of the medium (Wesley, 1996; Krewski et al., 2009). Interestingly, oral aluminum bioavailability is known to be increased by acidic pH, such as the pH in the human intestine, but in case of clinoptilolite tuff, it may be decreased, as this is a silicon-containing compound that releases certain amounts of dissolved silica (Jurkić et al., 2013). Data has been provided on the ability of silicon-rich mineral water or silicic acid to remove Al from the human organism (Buffoli et al., 2013; Davenward et al., 2013), and this Si and Al relation has been recognized as the main evolutionary mechanism for fighting ecotoxicity of aluminum in living organisms. Water-soluble silica forms may thus be acknowledged as important contributors to fighting aluminum detrimental effects on human and animal health, especially nowadays when the exposure to bioavailable free aluminum cation poses a serious problem due to industrial development (Exley, 2009; Beardmore et al., 2016; Exley, 2016).

In addition, we hypothesize that previously observed data on antitumor properties of clinoptilolite *in vitro* may be due to the activation of clinoptilolite surface by acids. Even though in the majority of *in vitro* studies, the cells were grown in micronized clinoptilolite pre-treated growth media, no ultracentrifugation was employed, which means that a colloid system containing finest clinoptilolite particles was used for experiments (Pavelić et al., 2001b; Katic et al., 2006). For instance, it is well-known that tumor cells have increased hydrogen peroxide levels that regulate specific signaling pathways and hydrogen peroxide may modify cysteine residues on antioxidative enzymes (Lennicke et al., 2015). Enzymes are deactivated during modification. Clinoptilolite can react with hydrogen peroxide (Canli and Abali, 2016), similar to other silica particles, and, in such situations, oxidative stress is induced either through the breakdown of hydrogen peroxides to hydroxyl radicals or through the breakdown of hydrogen peroxides and production of the hydroperoxyl radicals (Rochette and Vergely, 2008). Therefore, it is possible that the contact between clinoptilolite and tumor cells with increased hydrogen peroxide concentrations induces formation of free radicals; therefore, increases in the oxidative burden occur in tumor cells, which consequently die. Tumor cells are susceptible to increased oxidative stress and in our previous experiments, this effect was not visible or was lower in normal tested fibroblasts *in vitro* (Katic et al., 2006). Also, it cannot be ruled out that some clinoptilolite particles enter into tumor cells *in vitro*, as tumor cells are inherently depolarized (Yang and Brackenbury, 2013) and can uptake particles by endocytosis (Sincai et al., 2007). Recently, a new hypothesis has been suggested on the use of lipophilic anions that target cancer cells due to their distinct electrical properties (Forrest, 2015). As clinoptilolite particles are negatively-charged polyanions, they might also target cancer cells and induce additional oxidative stress upon entrance into the cytoplasm through hydrogen peroxide activation, increased production of ROS and its consequent depletion within the cell. The depletion of hydrogen peroxide and the increased ROS production during hydrogen peroxide reaction with a clinoptilolite surface may change the redox status of the cell, e.g., through inhibition of the transcription factor Nrf2. Indeed, in previous *in vitro* experiments on tumor cells, clinoptilolite antitumor effects were attributed to the modulation of the EGF-R, protein kinase B (PKB)/Akt, and nuclear factor kB (NfkB) signaling. They are interconnected with ROS and activity of Nrf2 (Pavelić et al., 2001b; Katic et al., 2006). This might be highly relevant for the survival of cancer cells as Nrf2 bears a proliferative role. In tumor cells, Nrf2 is usually activated by ROS-induced oncogenes, such as KRAS and c-MYC (DeNicola, 2011), and inhibition of its activity may contribute to the apoptosis of tumor cells and abrogated tumor growth (Ryoo et al., 2016).

## Clinoptilolite Toxicology in Animals and Humans

The basic structure of clinoptilolite is considered to be biologically neutral and non-toxic (Auerbach et al., 2003). EFSA recently released an expert opinion on the safety of natural zeolite clinoptilolite *in vivo* (EFSA Panel on Additives and Products or Substances used in Animal Feed, 2013). EFSA evaluated and proved the zeolite-clinoptilolite non-toxicity for animal feed at doses of 10000 mg/kg. Oral consumption of this type of zeolite, due to its extreme chemical stability, in EFSA’s opinion, does not represent a potential risk for *in vivo* applications (EFSA Panel on Additives and Products or Substances used in Animal Feed, 2013).

The first comprehensive acute, subchronic, and chronic toxicology evaluation of a clinoptilolite material *in vivo* was performed by Pavelić et al. (2001b). In this preclinical toxicology study, tribomechanically micronized clinoptilolite was evaluated at the ‘Ruer Bošković’ Institute in Zagreb, Croatia, according to the standards and regulations required at the time by the OECD. In that study, the effects associated with increasing exposure times were analyzed in three categories: (1) acute toxic responses up to 1 month in mice and rats, (2) subchronic toxic responses up to 3 months in mice and rats, and (3) chronic toxic responses up to 1 year in rats and 6 months in mice. Clinoptilolite was administered to the animals as a powder supplementing their usual diet. Toxicity studies were approached by setting a “limit” test, which means that high doses of the substance were applied during 15 or more days. Two doses were selected from the “limit” test, 400 mg/mice/day (3.2 times higher than the dose specified by the regulatory agency) and 1000 mg/mice/day (8 times higher). Recalculated from human use, they were 10 and 25 times higher than envisaged potential human exposure dosages (60 g/75 kg human body weight and 150 g/75 kg human body weight). The results showed that the “limit” test doses of the substance did not cause death for mice. Therefore, the “up and down” test on mice was performed with doses ranging from 60 to 400 mg/mice/day. Again, no toxicity was observed. Classical acute, subacute, and chronic tests on rats and mice were performed as well. Oral (in diet) administration to mice and rats showed no effects or changes that could be correlated to tribomechanically micronized clinoptilolite-supplementation. In addition, earlier in Pond and Yen (1983) published the first study on the clinoptilolite effects on the reproduction and progeny growth in rats with or without cadmium presence. They have shown protective effects of clinoptilolite on hematocrit and hemoglobin levels as well as on cadmium levels in the liver of pigs fed with cadmium in the presence of clinoptilolite in comparison to animals fed only with addition of cadmium to the diet.

Similarly, in another study performed by the European Union Cosmetic Ingredient Review Expert Panel, natural clinoptilolite showed no effects on female rat reproductive performance and it proved non-genotoxic in the Ames bacterial test system (Elmore, 2003). Moreover, in an independent study performed by Martin-Kleiner et al. (2001) effects of tribomechanically micronized clinoptilolite on the serum chemistry and hematopoiesis were evaluated in mice. The authors showed that the ingestion of clinoptilolite was well-tolerated and substantiated by unchanged body mass in clinoptilolite-supplemented mice. An increased level of potassium by 20% was detected in mice receiving the clinoptilolite-rich diet, while other changes in the serum chemistry were not observed. Erythrocyte, hemoglobin, and platelet levels in peripheral blood were not affected by clinoptilolite supplementation either.

Also, Muck-Seler and Pivac (2003) studied the effects of tribomechanically micronized and non-micronized clinoptilolite materials on the serotonergic 5-hydroxytryptamine receptors 5-HT(1A) and 5-HT(1B) in the brain of non-tumorous (control) and mammary carcinoma-bearing female mice. A reduced binding of 3[H]8-hydroxy-2-(di-n-propylamino)tetralin (3H-8-OH-DPAT) to 5-HT(1A) receptors in mammary carcinoma-bearing mice was normalized in animals supplemented by tribomechanically-micronized clinoptilolite. Also, the administration of clinoptilolite materials did not affect the binding of 3H—8-OH-DPAT to the studied receptors during prolonged administration. The authors speculated that the observed effects in tumor-bearing mice may be in correlation with the electrolytes balance, or immune system response to supplementation. A neuroprotective effect was also documented by Basha et al. (2013). Safety of the material was also proven by Ivkovic et al. (2004) where no adverse reactions to tribomechanically micronized clinoptilolite supplementation were observed in immunodeficient patients.

Some concerns were raised in public on the possible lead leakage from the natural clinoptilolite materials into the intestine. Still, extremely high affinity of clinoptilolite to lead has been documented previously, where sorption of lead and cadmium (Cd) on natural clinoptilolite was shown to be irreversible or very slowly reversible (Hamidpour et al., 2010), and, in particular, it was shown to be high in an acidic environment (Perić et al., 2004). These results were obtained in very simple *in vitro* models that may not adequately mimic human digestion. Furthermore, a high capacity of zeolite lead adsorption occurs in the pH range 3–11 (Payne and Abdel-Fattah, 2004) and the leaching of lead from lead-preloaded clinoptilolite occurs mainly in pH under 1, which is not relevant to conditions in the human body, as shown by Petrakakis et al. (2007). The authors conducted the study according to the standard procedures, Toxicity Characteristic Leaching Procedure/Environmental protection agency/Resource Conservation and Recovery Act (TCLP/EPA/RCRA) (1311), EPA Methods 1310, 1320 and DIN 38414-S4, and provided evidence of the pH being the main factor affecting Pb leaching from clinoptilolite. Interestingly, in the pH 3 and higher Pb, the leakage was less than 1%, while at pH 1 the leakage was observed up to 20% of the initial lead content. Furthermore, the authors show that the re-adsorption of Pb particles that leach from the solid material may occur as well; for lead this process occurred at pH 1.5 and 2. The Pb leaching percentage may, in the authors’ opinion, be generally correlated with an increasing initial load but is not affected by the agitation rate or particle size. Also, previously published results from trials on animals and human subjects showed a strong clinoptilolite detoxifying effect and reduction of Pb content *in vivo*. For instance, tissue lead concentrations in lead-intoxicated rats with or without clinoptilolite supplementation clearly show that Pb concentrations were not increased in animals fed with clinoptilolite and that the intoxication burden in animals can be even alleviated by clinoptilolite supplementation (Beltcheva et al., 2012, 2015; Basha et al., 2013a). Similarly, in the study by Fokas et al. (2004), clinoptilolite was added to the diet of growing pigs at 20 g/kg and no significant increase of Pb concentration in blood and edible tissues was measured. In this study, however, Pb levels were not discussed in the context of stored Pb levels in the bones and Pb levels in the bones were not assessed. This is why definite conclusions on eventual lead fate in the blood and organism of animals fed with clinoptilolite supplemented diet in this study cannot, be conclusive. Moreover, a clinical study comprising 22 human subjects evaluated the effects of clinoptilolite treatment on chronic diseases which could be traced back to heavy metal poisoning. During treatment with activated clinoptilolite from 7 to 30 days in total, both urine and blood serum were collected and tested for heavy metals and electrolytes. In this study, the daily intake of activated clinoptilolite suspension was effective in removal of toxic heavy metals from the body *via* urine (Flowers et al., 2009). Urine is, indeed, important in elimination of lead released from the bones or body compartments, i.e., in chelation therapy where upon quenching of lead from different sites of the body it is expelled through urine (Flora et al., 2012). The high lead leakage from the material into the body is theoretically possible but this would eventually happen from tuff materials with extremely high content of lead where the theoretical absorption would be subject to many different physiological parameters and health conditions. Another clinical study on human subjects showed detoxifying effectiveness of clinoptilolite. A total of 102 heavy-metal contaminated men were investigated and decreased concentrations of harmful metals (Cd, Pb, Cu, Cr, and Ni) were measured in their hair after a 30-day supplementation with clinoptilolite. This decrease in harmful metal concentrations was a result of the clinoptilolite detoxification function and probable restoration of the body mineral metabolism homeostasis (Zhakov, 2003). Importantly, while great danger exists in removing the physiologically important electrolytes from the serum in a classical detoxification process, this has not been observed in clinoptilolite trials both in humans and animals, where no substantial changes in physiologically relevant trace elements or vitamins were observed even after long-term administration (Papaioannou et al., 2002; Katsoulos et al., 2005b; Flowers et al., 2009).

In conclusion, clinoptilolite materials tested in the scientific literature proved to be generally safe for *in vivo* applications even though each material seems to retain its own physical-chemical characteristics and exerts specific biological effects that cannot be readily transferable to other materials. Different particle sizes, surface areas, and cation compositions may induce different biological effects and exert different levels of effectiveness. Biological effects and toxicology data should therefore be carefully evaluated according to the type of clinoptilolite material or clinoptilolite-based preparations used in a particular study or application. In this paper, the cited literature on clinoptilolite effects *in vitro* and *in vivo* provides data for clinoptilolite materials (tuffs) from different sources/continents, of different purity, chemical composition, and that were prepared for oral application by use of different milling processing methods. Moreover, the research goals and experimental designs were different. This is why no absolute generalization on the mechanisms of action for clinoptilolite materials (tuffs) may be done at this point. Still, presented studies provide intriguing data on positive medical effects for this type of materials, especially effects on the immune system and detoxification, all substantiated by so far presented safe profile. In the future, it would be highly helpful to gather scientific data on the direct relationship between specific clinoptilolite material properties and sources with positive or negative effects and mechanisms of action *in vivo*. This will fill in the current gaps in research presented so far as similarly suggested by Colella (2011). Collela also emphasized the variability and heterogeneity of the clinoptilolite material used in different applications and studies and suggested to study in detail the applications and mechanisms of clinoptilolite materials in light of known and well-established properties or behaviors.

## Conclusion

In agreement with the scientific evidence presented in the literature so far, it can be generally stated that clinoptilolite-based materials, including the so-called activated materials, may be regarded as safe for *in vivo* consumption. A variety of highly positive effects on animal and human health were documented thus far for clinoptilolite-based materials. Due to clinoptilolite’s remarkable ion-exchange and adsorption properties and consequent detoxifying effects, it has proven useful in the elimination of a variety of contaminants from the body or in amelioration of the intestinal status. An indirect systemic detoxification effect attributed to clinoptilolite-based material supplementation in the diet of both animals and humans was documented in other organs as well, e.g., liver. However, the observed positive systemic mechanisms are still not completely understood. We hypothesize that they may be at least partially attributed to the restoration of the human homeostasis due to local detoxification properties within the intestine, the release of dissolved silica forms from the clinoptilolite tuff that enter from the intestine into the blood, as well as to clinoptilolite’s immunomodulatory effects. The observed local immunomodulatory effects of clinoptilolite involve the induction of immune responses through Peyer’s patches and/or possible positive effects on microbial intestinal populations through still unknown mechanisms. These local effects may have a systemic ‘echo’ on the whole immune status as well, as observed in some studies.

Finally, clinoptilolite’s antioxidant effects and restoration of antioxidant defense mechanisms may also be linked to the positive general systemic impact. However, conclusive statements on the exact applications and benefits of clinoptilolite-based materials in humans should be carefully investigated and analyzed for each specific clinoptilolite material as the mechanisms of action may have correlations with the specific material’s physical and chemical properties. Currently, different clinoptilolite-containing materials are used in medical applications worldwide. These materials contain different percentages of clinoptilolite and different compositions. Also, clinoptilolite-containing natural tuffs come with small quantities of other trace elements, and clinoptilolite is always pre-loaded with various cations. Some of the alkaline ions contained in the crystal lattice, mainly Na^+^, Ca^2+^, Mg^2^, and K^+^, may be readily released during the ion-exchange process. While these parameters may not be that relevant for agricultural or industrial applications, veterinary and human applications would require a higher level of control *via* a quality control system in the production, both of the raw material and the final products. For example, a proper mining process with adequate cleaning, sieving, de-hydrating, and pre-milling processes, along with elemental and microbiological examination of the clinoptilolite materials, might be considered among essential requirements for ensuring purity and quality (in relation to the high clinoptilolite content in the tuff) of the final materials for *in vivo* consumption.

## Author Contributions

SKP generated the main idea and wrote the manuscript, generated and shaped presented hypotheses, performed literature search and analysis, prepared the figures and tables, discussed and systematized all literature data. JSM prepared parts related to clinical aspects of clinoptilolite effects *in vivo*, was involved in the preparation of the table. DG performed the literature search, participated in writing of the manuscript related to oxidative stress and immune system, and participated in shaping of the hypothesis of zeolite molecular effects *in vivo*. AF performed literature search on physical-chemical properties of clinoptilolite and wrote parts of the manuscript related to clinoptilolite chemistry. NP performed a critical review of data and literature, edited the paper content and its final content. KP performed literature search related to clinical aspects and toxicology, discussed clinical aspects, and helped to draft the manuscript.

## Conflict of Interest Statement

The authors declare that the research was conducted in the absence of any commercial or financial relationships that could be construed as a potential conflict of interest.

## Abbreviations

5-HT(1A) and 5-HT(1B): serotonergic 5-hydroxytryptamine receptors in the brain
3H-8-OH-DPAT: 3[H]8-hydroxy-2-(di-n-propylamino)tetralin
Al: aluminum
Al^3+^: aluminum(III)-cation
ALT: aspartate aminotransferase
AST: alanine aminotransferase
Ba: barium
Ca: calcium
Co: cobalt
CO_2_: carbon dioxide
Cd: cadmium
Cr: chromium
Cs: caesium
Cu: copper
EDTA: ethylenediaminetetraacetic acid
EFSA: The European Food Safety Authority
EGF-R: epidermal growth factor receptor
Fe: iron
GALT: gut-associated lymphoid tissue
GGT: gamma-glutamyl transferase
GSH: glutathione
HEU: clinoptilolite
Hg: mercury
H_2_S: hydrogen sulfide
H_2_O_2_: hydrogen peroxide
K: potassium
Mn: manganese
MALT: mucosa-associated lymphoid tissue
MDA: malondialdehyde
Na: sodium
NfkB: nuclear factor kB
Ni: nickel
$O_{2}^{•-}$: superoxide anion
OECD: Organization for Economic Cooperation and Development
⋅OH: hydroxyl radical
Pb: lead
(PKB)/Akt: protein kinase B/Akt kinase
PMA: micronized clinoptilolite material
Prx: peroxiredoxin
ROS: reactive oxygen species
Si: silicon
SOD: superoxide dismutase
Sr: strontium
TCLP/EPA/RCRA: Toxicity Characteristic Leaching Procedure/Environmental protection agency/Resource Conservation and Recovery Act
Trx: thioredoxin
Zn: zinc
